# Productivity benefits of warming at regional scale could be offset by detrimental impacts on site level hydrology

**DOI:** 10.1038/s41598-017-15136-8

**Published:** 2017-11-09

**Authors:** Qing Zeng, Yamian Zhang, Li Wen, Zhaxijie Li, Hairui Duo, Guangchun Lei

**Affiliations:** 1 School of Nature Conservation, Beijing Forestry University, Beijing, China; 2 Science Division, Office of Environment and Heritage, Sydney, New South Wales Australia; 3 Tibet Museum of Natural Science, Lhasa, China

## Abstract

Climate change affects the distribution and persistence of wildlife. Broad scale studies have demonstrated that climate change shifts the geographic ranges and phenology of species. These findings are influential for making high level strategies but not practical enough to guide site specific management. In this study, we explored the environment factors affecting the population of Bar-headed Goose in the key breeding site of Qinghai using generalized additive mixed model (GAMM). Our results showed that 1) there were significant increasing trends in climate variables and river flows to the Qinghai Lake; 2) NDVI in the sites decreased significantly despite the regional positive trend induced by the warmer and wetter climate; 3) NDVI at site scale was negatively correlated to lake water level; and 4) the abundance of Bar-headed Goose decreased significantly at all sites. While the abundance was positively related to NDVI at breeding sites, the GAMM revealed an opposite relationship at foraging areas. Our findings demonstrated the multi-facet effects of climate change on population dynamics; and the effect at global/regional scale could be complicated by site level factors.

## Introduction

Clear evidences show that anthropogenic global warming has already affected biodiversity at individual, species, population, community, ecosystem and biome scales^1,2^. As one of the most serious and widespread threats^3^, the continuously warming may have already resulted in several recent species extinctions^4^, and the sixth mass extinction in the history of the earth was projected^5,6^. Migratory birds are expected to be among the most affected species group^7^, with response involving changes in distribution, habitat selection, migratory behaviour, and phenology or the timing of breeding^8,9^. The ecologically specialized or range-restricted species, particularly polar and alpine species, show more-severe range contractions than other groups, and have been the first groups in which whole species have gone extinct due to recent climate change^10^.

The increase rate of annual temperature is positively correlated with elevation^11^. Compared with the global average, the warming of the Qinghai-Tibetan Plateau occurred earlier, and the rate of temperature increase exceeded those at the same latitudinal zone during the same period^12^. Moreover, known as the “Asian Water Tower”, there are 36,800 glaciers on the Qinghai-Tibetan Plateau covering an area of 49,873 km^2,13^, which makes it the largest ice mass outside the polar regions. The unique geographic setting makes Qinghai-Tibetan Plateau one of the most vulnerable areas to respond to global climate change^14^.

The wild Bar-headed Geese (*Anser indicus*) are endemic upland grazer found exclusively in central Asia^15^. The geese breed on a variety of wetlands in highland plateaus of central Asia and winter in lowland swamps, lakes and rivers in China from southern Tibet east to Guizhou, and from Pakistan east to Myanmar^15^. As one of the highest and most iconic trans-mountain migratory species in the world^16^, its migration includes crossing a broad front of the Himalaya Cordillera, a significant formidable barrier to avian migration^17^. While there are general increases in wild goose populations since the 1960 s due to global conservation efforts^18^, the Bar-headed Geese may be vulnerable to population decline resulting from habitat loss in over-wintering areas^19^ and outbreak of avian influenza^20^. Being one of major breeding sites for Bar-headed Geese, habitat alteration such as vegetation composition change around Qinghai Lake induced by severe climate change^21^ may be one of the reasons underpinning this opposite population dynamics. Qinghai Lake has experienced significant changes including water level and water quality during the past three decades and is still changing rapidly due to climate change^22^. These changes may have profound impacts on the breeding population of Bar-headed Goose by influencing food quality or availability^23^.

**Figure 1:**
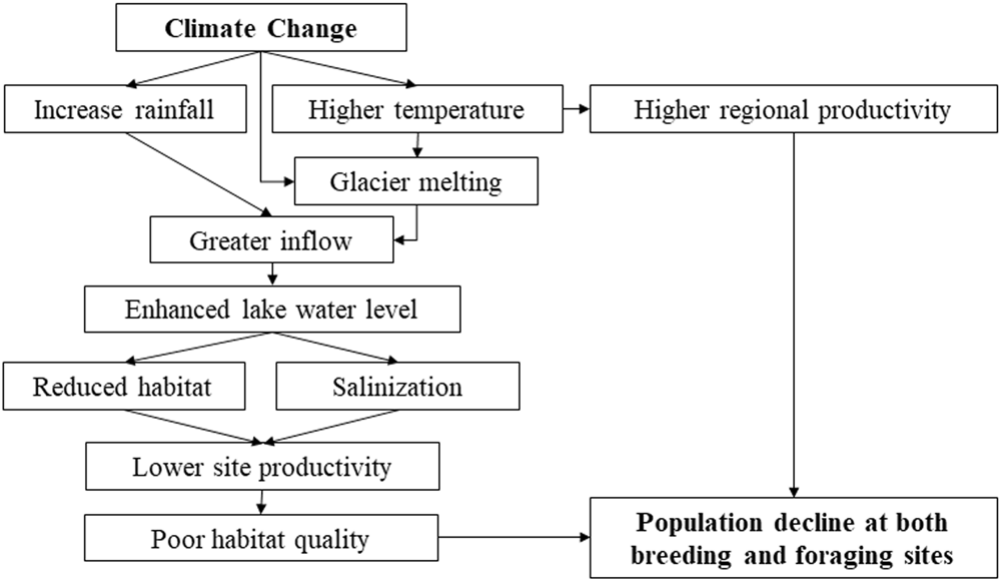
Flow chart shows the rational of the study and working hypotheses.

Progress toward understanding the effects of climate change on bird population, has been challenging, because non-climatic influences such as hunting and harvesting^24^, generally dominate local, short-term biological changes^1^. The breeding population in Qinghai Lake provides a unique opportunity to study the demographic effects of climate change. First, as Buddhism is the dominating religion of the main distribution region, the pressure of wildlife hunting and harvesting are expected to be low. Second, due to the high altitude and low human population density, the region is relatively free of development and lack of modern agricultural activity, thus in a relatively natural condition. Third, at their wintering sites, the bar-head geese feed on cultivated lands, in which the conditions such as food abundance are relatively stable comparing to natural wetlands. Thus, the influences of wintering sites might be smaller than those of breeding grounds. In this study, we first explored the long-term (1958–2016) climatic trends and their impacts on Lake hydrology and lake shore habitat quality using time series models. The Moderate Resolution Imaging Spectroradiometer (MODIS) normalized difference vegetation index (NDVI) was used as proxy of habitat quality. We then modelled the spatial and temporal variations of Bar-headed Goose population dynamics (2007–2016) at the key breeding sites around the Qinghai Lake within the generalised additive mixed modelling framework (GAMM^25^). We test the following hypotheses (Fig. 1) concerning the effects of climate change in Bar-headed goose population: 1) that the productivity benefits of global warming at regional scale^26^ is offset by the detrimental impacts on site level hydrology through habitat loss and degradation; 2) due to distinct dispersal capacity and mobility^27^, the relationship between bird abundance and environmental drivers at breeding sites may be divergent from foraging sites.

## Results

### Spatial and temporal distribution of *Anser indicus*

The bird survey data showed that the bar-headed geese used Dandao (S04) and Sankuaisi (S19) as the main breeding grounds and the other sites as foraging areas. There were considerable spatial and temporal (between year) variations for both breeding grounds and foraging areas (Supplementary Figure S1). During the peak breeding month of May, the majority of birds congregated at S04 and S19 (Supplementary Figure S1, left). After the chicks fledged, they dispersed to other surrounding areas for foraging and very few birds were observed remaining at its breeding grounds (Supplementary Figure S1, right).

### Long-term Trends in climate and hydrology

The long-term trends in climate and hydrology in Qinghai Lake was summarized in Table 1. All the tested variables had significant annual increasing trend (p < 0.001, Table 1). Furthermore, with only three exceptions (i.e. rainfall in July, inflow in Aug and Sep), most of the variables showed a significant increasing trend for each month (Table 1).

**Table 1:** Summary of the Correlated Seasonal Mann-Kendall Tests for the long-term trends in climate (1958–2015), inflow and Lake water level (1995–2015), and NDVI (2000–2015) in Qinghai Lake region. Bold indicates no trend was detected.

	Rainfall		Temperature		Flow		Water Level		NDVI*	
Month	Z	P^^^	Z	p	Z	p	Z	*p*	Z	p
Jan	5.289	0.000	2.992	0.003	2.839	0.005	2.295	0.022	−1.831	0.067
Feb	6.064	0.000	4.394	0.000	2.174	0.030	2.297	0.022	−3.118	0.002
Mar	4.917	0.000	2.160	0.031	1.329	0.184	2.176	0.030	−2.722	0.006
Apr	4.878	0.000	2.489	0.013	3.563	0.000	2.150	0.032	−2.029	0.042
May	2.972	0.003	2.791	0.005	3.201	0.001	2.266	0.023	−0.445	**0.656**
Jun	2.288	0.022	4.790	0.000	2.235	0.025	2.174	0.030	−0.643	**0.520**
Jul	1.046	**0.295**	2.999	0.003	2.295	0.022	2.295	0.022	−1.633	**0.102**
Aug	2.080	0.038	2.455	0.014	1.389	**0.165**	2.602	0.009	−1.227	**0.126**
Sep	2.053	0.040	4.314	0.000	1.570	**0.116**	2.537	0.011	−0.940	**0.347**
Oct	4.403	0.000	3.488	0.000	1.813	0.070	3.051	0.002	−2.524	0.012
Nov	4.900	0.000	3.555	0.000	2.689	0.007	3.204	0.001	−4.008	0.000
Dec	3.974	0.000	2.703	0.007	3.201	0.001	3.172	0.002	−2.128	0.033
Overall	6.300	0.000	6.000	0.000	3.600	0.000	2.600	0.009	−3.400	0.001

*Spatial mean for Dandao (S04, Fig. 6); ^^^0.000 indicates <0.001.

**Figure 2:**
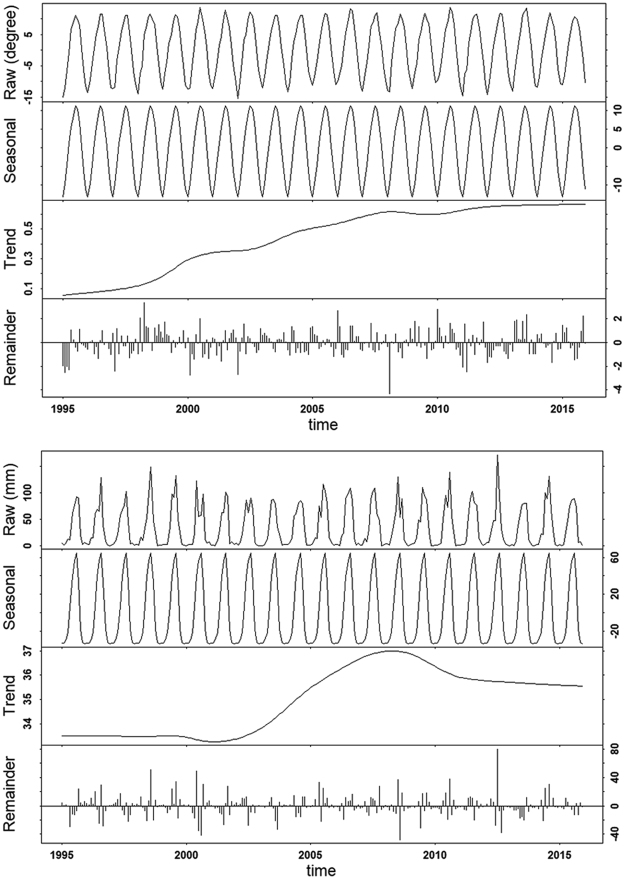
Seasonal cycles and trends of monthly mean temperature (upper) and rainfall (upper) in Qinghai Lake for the period of 1995–2015. The raw monthly records were decomposed in to seasonal cycles and long-term trend using weighted scatterplot smoothing.

For the monthly mean temperature, the increase was significant for each month, and the largest increase occurred in June and February (Table 1). Monthly mean temperature was the only variables showed a monotonic increasing trend during the whole period although the increasing was more rapid before 2010 (upper panel, Fig. 2). For rainfall, there was significant increase in each month except July, when the increase was not significant (p = 0.295 for July, Table 1). In addition, the Z values indicates that there were larger increases in winter, spring and autumn than in summer; and the largest increase occurred in January and February, the coldest months (Table 1). The long-term trend analysis indicated that there were three phases of changes in rainfall (lower panel, Fig. 2): there was little changes for 1995 to 2001; from 2002 to 2010, there was a rapid increase; and for the period of 2011–2015, there was little inter-annual change.

**Figure 3:**
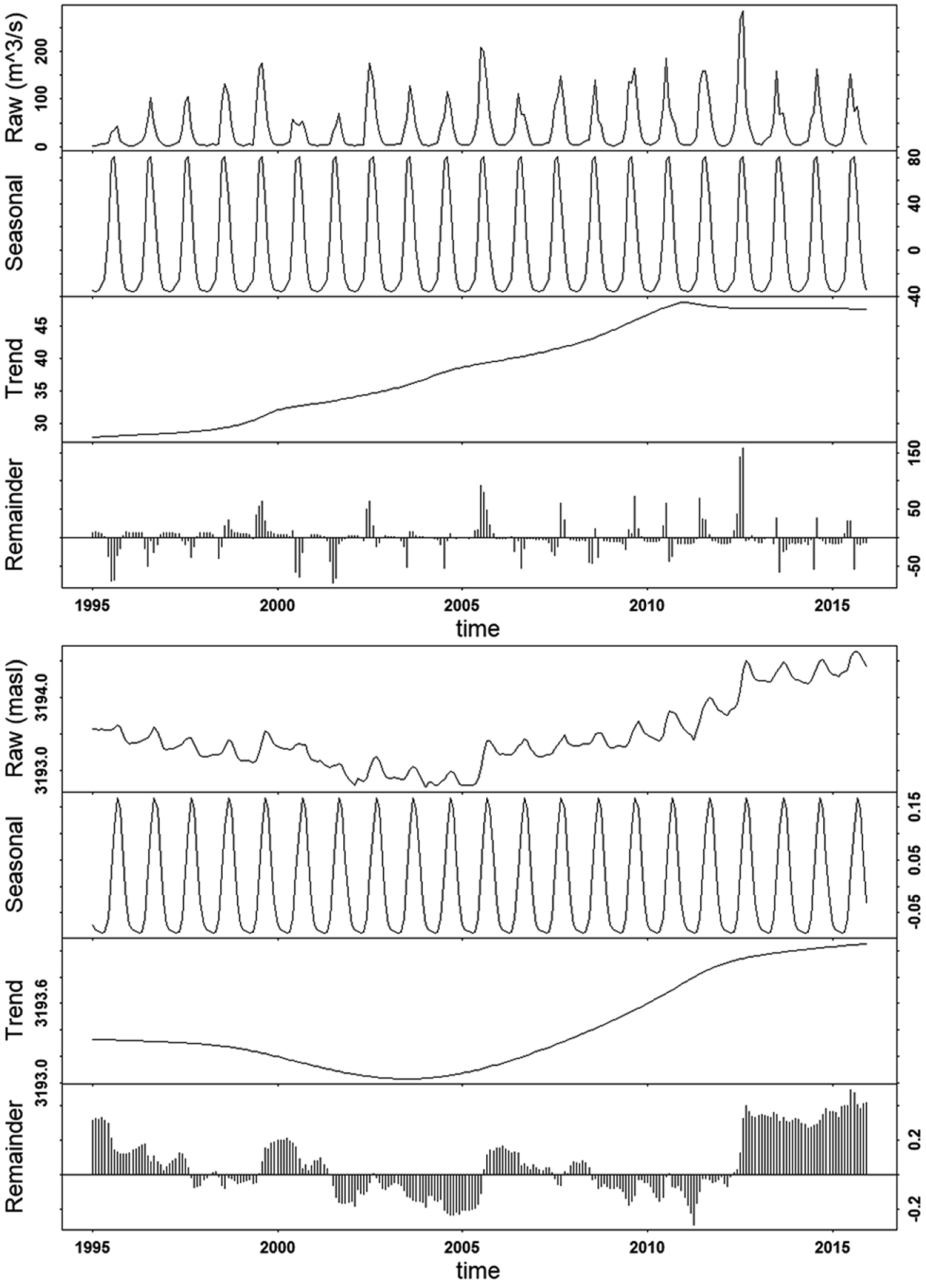
Seasonal cycles and trends of monthly mean inflow (upper) and lake water level (lower) in Qinghai Lake for the period of 1995–2015. The raw monthly records were decomposed in to seasonal cycles and long-term trend using weighted scatterplot smoothing.

The flows to Qinghai Lake also increased significantly for the period of 1995–2015, and significant increase was found in every month except August and September (Table 1). Similar to rainfall, the increase in river flow was larger in the colder months; and the largest increase of flow occurred in April, corresponding to the onset of de-frozen in this region. The long-term trend analysis (upper panel, Fig. 3) showed that the river flow continuously increased till 2011, after which there were little inter-annual variations.

The lake water level had also increased significantly for the period of 1995–2015; and the increase was significant for each month (Table 1). However, compared to climatic variables and river flow, which had a general increasing trend for the investigated period, the water level showed a clear turning-point around 2004, before which the lake water level decreased constantly, but increased consistently since (lower panel, Fig. 3).

### Spatial and temporal variations in NDVI

For the majority of sites, the spatial mean of monthly NDVI showed an overall decreasing trend for the period of 2001–2015 (Table 1). The Correlated Seasonal Mann-Kendall Test indicated that the decreasing was significant (p = 0.0001, Table 1). However, the CSMK test showed that there was not significant change for the hotter months (i.e. from May to September, Table 1).

Table 2 summarised the results of the final GAMM for spatial mean NDVI for the main bar-headed goose habitat sites in Qinghai Lake region. All models had high to very high accuracy (adjusted *R* ^2^ ranged from 0.839 to 0.947, except S19 with a moderate adj-*R* ^2^ of 0.611 (Table 2). S19 is an island which is subjected to inundation during the high water seasons. The NDVI showed a strong seasonal cycle peaked at August (p < 0.0001, Table 2, Supplementary Figure S2).

**Table 2:** Summary of the GAMM for spatial mean monthly NDVI at nine main habitat sites. The terms included in the final model, their p-values (Bold indicate significant at 0.1 level) and estimated degrees of freedom (EDF) were listed. The results for autoregression were not shown for simplicity.

Site	*s*(Month)	*s*(Time)	*s*(MeanT)	*s*(WL)	*s*(Rainfall)	Adj-*R* ^2^
S01	**0.000** (10)			**0.000** (1.00)		0.839
S04	**0.000** (10)	**0.075** (1.00)		**0.000** (1.00)		0.926
S07	**0.000** (10)	**0.000** (4.23)		**0.081** (1.00)		0.947
S09	**0.000** (10)	0.113 (4.04)		**0.001** (5.03)		0.929
S12	**0.000** (10)		0.183 (1.00)	**0.000** (1.82)	**0.009** (1.00)	0.922
S17	**0.000** (10)	0.135 (3.70)	0.126 (1.00)	**0.000** (1.00)	0.179 (1.00)	0.949
S19	**0.000** (10)		**0.003** (1.00)	**0.036** (3.45)		0.611
S22	**0.000** (10)	**0.000** (6.63)	**0.072** (1.00)	**0.000** (4.72)		0.854
S23	**0.000** (10)	**0.000** (3.82)	**0.079** (2.40)		**0.028** (1.00)	0.951

0.000 indicates p < 0.001; Rainfall and MeanT are the total monthly rainfall and mean monthly temperature; Month is the calendar month; Time is the number of month since February 2000 when the MODIS NDVI started; WL is the Lake Water level; Adj-*R* ^2^ is a smooth function.

**Figure 4:**
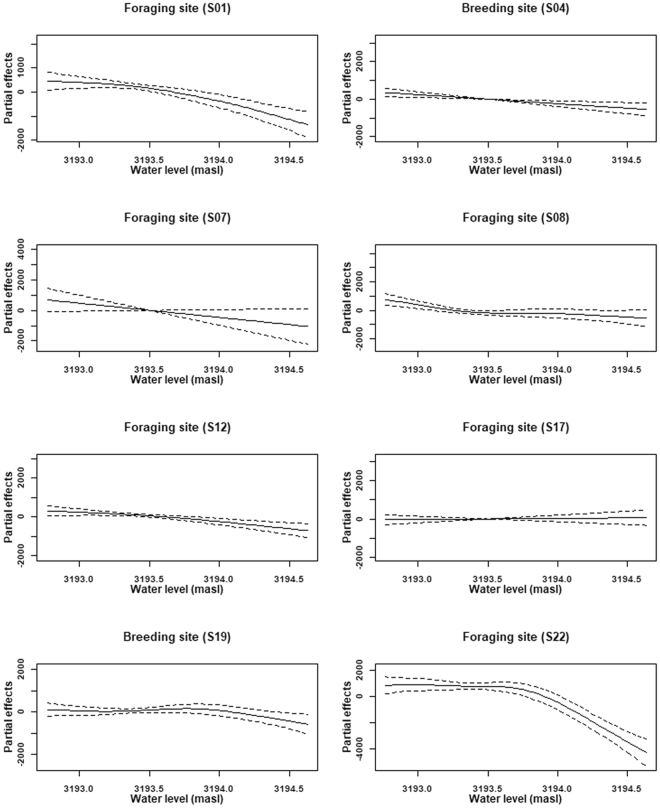
The partial effect of lake water level (masl, meter above sea level) on spatial mean NDVI for the main bar-headed goose habitat in Qinghai Lake. Dotted lines are standard errors.

The lake water level had significantly negative impacts on NDVI for eight out of the nine sites (Table 2, Fig. 4). The effect of water level on NDVI was largely linear for most of the sites indicated by the estimate degree of freedom (EDF, close to 1 means linear, the larger the EDF, the more nonlinear the effect^25^). For S22, the modelled effect curve indicated that there was threshold around 3194 masl, above which the NDVI decreasing rapidly (Fig. 4).

### The response of *Anser indicus* population to the changes in habitat quality

The final population GAMM included the random terms for Site, and random smooth for NDVI and Time by habitat types (i.e. breeding or foraging); and all other climatic (temperature and rainfall) and hydrological (lake water level) was not included (Supplementary Table S1). The model had relatively high performance with an adjusted *R* ^2^ of 0.898 and explained 67.4% of the total deviance in the observed population data.

**Figure 5:**
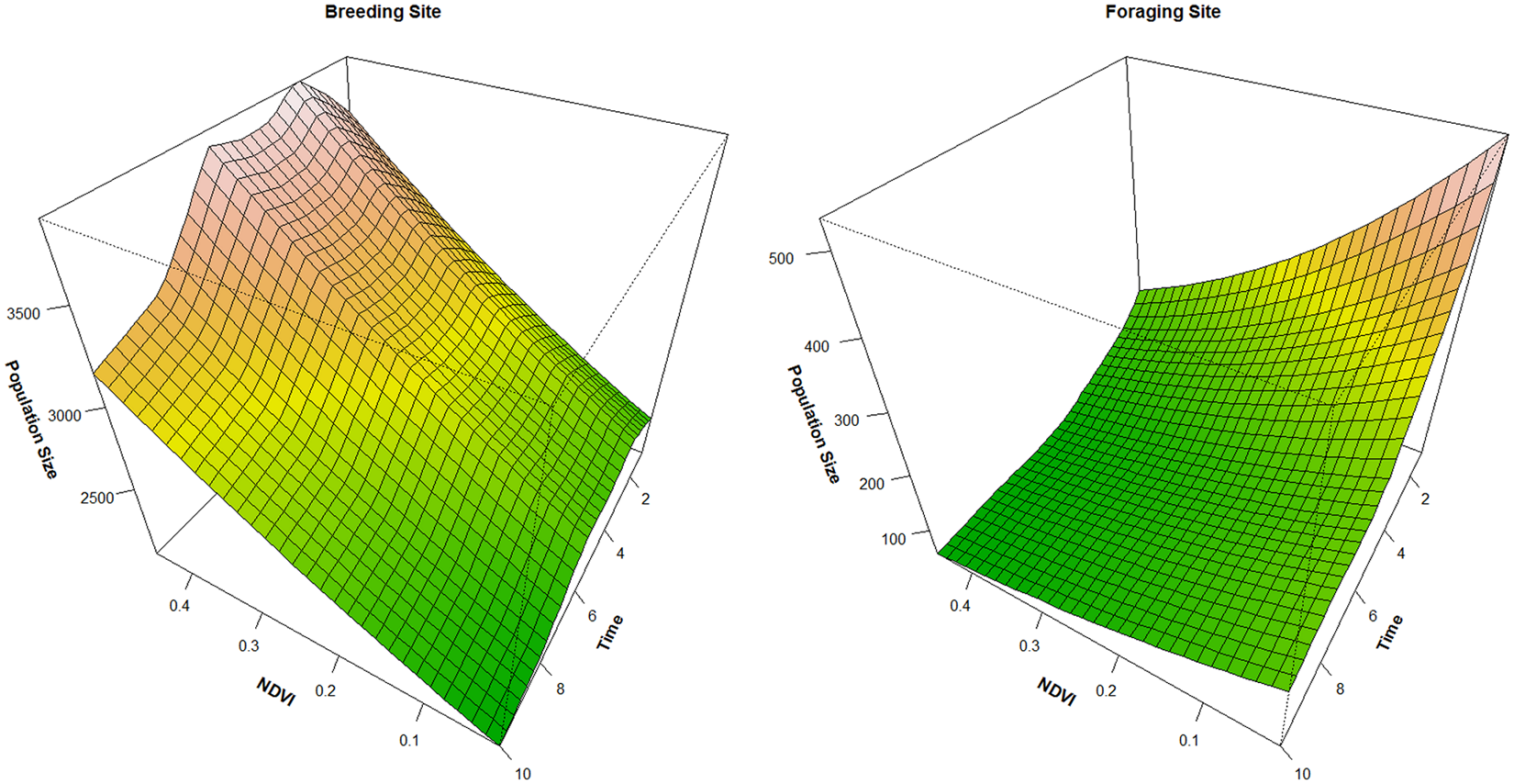
The response surface of bar-headed geese to NDVI and Time at breeding grounds (left) and foraging sites (right).

As expected population size varied significantly among survey site (p < 0.001, Supplementary Table S1). The effect of environmental factors on the population varied between the breeding grounds and foraging areas (Supplementary Table S1 and Fig. 5). At the breeding grounds, the effects of NDVI was significant and generally linear (p < 0.000, EDF = 0.867, Fig. 5). The population size increased linearly with NDVI; and decreased nonlinearly with time (left, Fig. 5). The decreasing in population started from 2010 and continued till the end of study period (i.e. 2016). In addition, the effect of NDVI seemed to be larger than that of time (left, Fig. 5). At the forage sites, the decisive factor was time, indicated by the modelled response surface (right, Fig. 5). The nonlinear effect curve of Time (right, Fig. 5) indicated that the decreasing trend slowed down from around 2010 and kept relatively stable since. On contrast to the breeding ground, the effect of NDVI was negative even though it was small (compared with the decreasing trend). The comparison of the two response surfaces (Fig. 5) suggested that NDVI had a more important role in determining bar-headed goose population at breeding site than at foraging site.

## Discussion and Conclusion

There are ample evidences suggesting that there is a general increasing trend in the range and abundance of wild geese throughout the northern hemisphere since 1960s^28^. However, our multi-year survey of the bar-head goose at its major breeding sites showed a declining trend. This discrepancy of local variation from global trend implies environmental changes at local scale may alter the ecological characters of its breeding habitat, and become unfavourable for the reproduction of this species.

### Water level change in Qinghai Lake

Our study showed an increasing trend of water level in Qinghai Lake for the period of 2005 to 2015, which is consistent with a study on ICESat laser altimetry and *in situ* measurements for the period of 2003 to 2009^29^. In recent years, 68.6% of large lakes (>100 km^2^)^30^ and 89% of the salt lakes^31^ in Qinghai-Tibetan Plateau have experienced water level rising. The warmer ambient temperature and changes in regional moisture^32^ contribute to rainfall increase, which in turn lead to the elevated river flow. In addition, a warmer climate results in melting of glaciers and delaying ice formation^27^. There is increasing evidence that over two-thirds of glaciers in the Hindu Kush-Himalayan-Tibetan (HKHT) region are retreating more rapidly since the 1950s (IPCC, 2007), which also has positive effects on river flow^33^. However, our trend analysis results showed a constant decreasing trend in lake water level from 1995 to 2005 although at a much lower rate. This decrease in water level was also observed in Zhang, *et al*.^29^. The opposite directions of trend in 2005 could not be explained by the long-term changes in climate (temperature and rainfall) and river flow, which showed a more persistent increasing trend for the studied period, even though there were phases when trends were absent (e.g. rainfall was rather stable in periods of 1995–2001 and 2011–2015). Evaporation, which also increased in Qinghai region^29^ with the warmer climate, could have a role in this disagreement; and a detailed water mass balance analysis is beyond the scope of this study.

### Impacts of Water Level on mean NDVI

NDVI, often referred as a proxy for vegetation photosynthetic activity, offers an integrated signal for exploring the climatic, hydrological and anthropogenic influences. In the study area, where the major vegetation types are swamp meadows and alpine meadows in semi-arid alpine continental climate, NDVI would increase with temperature and rainfall as demonstrated in other studies^26,34,35^. The partial effect curves of the final GAMM also showed the positive effects of temperature and rainfall on NDVI for models included the variables. Nevertheless, with the positive trends in both temperature and rainfall confirmed, the spatial mean NDVI in six of the nine major habitats showed a decreasing trend, implying other environmental factors may also take effects in the condition and state of vegetation community.

Water level is regarded as an important factor for the structure and function of lake ecosystem^36^, especially for the littoral zone of large deep lakes^37^. A small changes of water level may result in a large shift in plant communities as well as their productivity^36^. We found significant decline in NDVI time series for points that were not inundated using CMSK test despite the general increasing trend for the region^34,35^. The GAMM results indicated that the spatial mean NDVI was negatively related to lake water level when the long-term trend and seasonality were accounted for. These results gave a strong indication that the benefits of climatic change (i.e. warmer and wetter climate) was offset by water level raising. Lake water level raising influences littoral vegetation community in two ways. First, the raising water level would inundate part of land surface, directly reduce the size of vegetation patches resulting in a smaller spatial mean NDVI. Second, Qinghai Lake is saline with salinity level of 12.4 g/L^38,39^, the raising water level could induce the salinization of littoral zone, negatively affect plant growth. Although we have not monitored the vegetation community changes over the study period, the current dominated species such as such as *Suaeda glauca* and *Puccinellia distans* can tolerate high salinity providing circumstantial evidence of soil salinization.

### Habitat Quality and Bar-headed Goose population trend

Climate has a profound effect on species distributions, and our results show that this is also applicable for the distribution and population dynamics of Bar-headed Goose. For many species, the primary impact of climate change may through effects on synchrony with food, changing the timing and abundance of food supplies and other resources^40^. While climate warming may have some beneficial effects on migratory birds, e.g. earlier spring hatching and longer breeding season^41^, negative effects are also possible, e.g. mistiming with food supply^7^, less suitable habitats available. Climate change related influences to Qinghai Lake are predicted to be particularly dramatic^42^. Our results suggested that the destructive impacts might excess the positive effect as response surface of the final GAMM showed clearly a decreasing trend in bird populations at both breeding and forging sites.

Although none of the climatic and hydrological variables was included in the final model, their effects on Bar-head Goose were realized through their impacts on NDVI, which was significantly related to the observed bird abundance. The elevated lake water level induced the degradation of habitat quality (i.e. decreased NDVI in this study), causing the Bar-headed Goose to switch to shallower and more suitable sites. Furthermore, the final model suggested the divergent responses to both time and NDVI for breeding and foraging sites. At the breeding sites, the positive effects of NDVI seemed to be more extensive indicated by the steeper upwards slope, and the decline was slower shown by a non-linear and flatter slope. There are a few likely reasons for the greater importance of NDVI at breeding sites. First, breeding is an energy demanding task. Birds need more food during the breeding season and food availability influences multiple stages of the breeding cycle of birds. Second, animals are general less mobile during breeding^27^, and the new nestlings have limited dispersal capacity. Therefore, sites with sufficient food and high supply/demand ratio benefit breeding success in many ways, including the advancements of laying^43^, shorter incubation periods^37^, greater hatching success^44^, and larger clutch size^45^ and higher fitness of breeders and nestlings^38^.

Beside the importance of food availability and quality, other factors such as predator abundance and nest concealment^46^, site fidelity as well as social cues^44^ are also critical in bird breeding site selection. In other words, because of the limitation in alternatives, the Bar-headed geese may still select the same sites to build their nests even through the site is becoming smaller and of poorer quality (in term of food abundance as indicated by the decreasing in spatial mean NDVI). This may partially explain the slow and lagged decline in bird abundance at the breeding sites. On contrast, the foraging pattern of an animal, especially large grazing bird as the Bar-headed goose, mainly depends on the abundance and accessibility of food^25,47^. A warmer and wetter climate may bring better and richer food resources elsewhere in the region^26^. With their high dispersal capacity^48^, the Bar-headed Goose may explore a wider area when the food availability becomes lower at the littoral zone of Qinghai Lake. The sharp decreasing curve modelled for the foraging sites may reflect the broader distribution rather than a decline in the population.

## Data and Methods

### Study site

**Figure 6:**
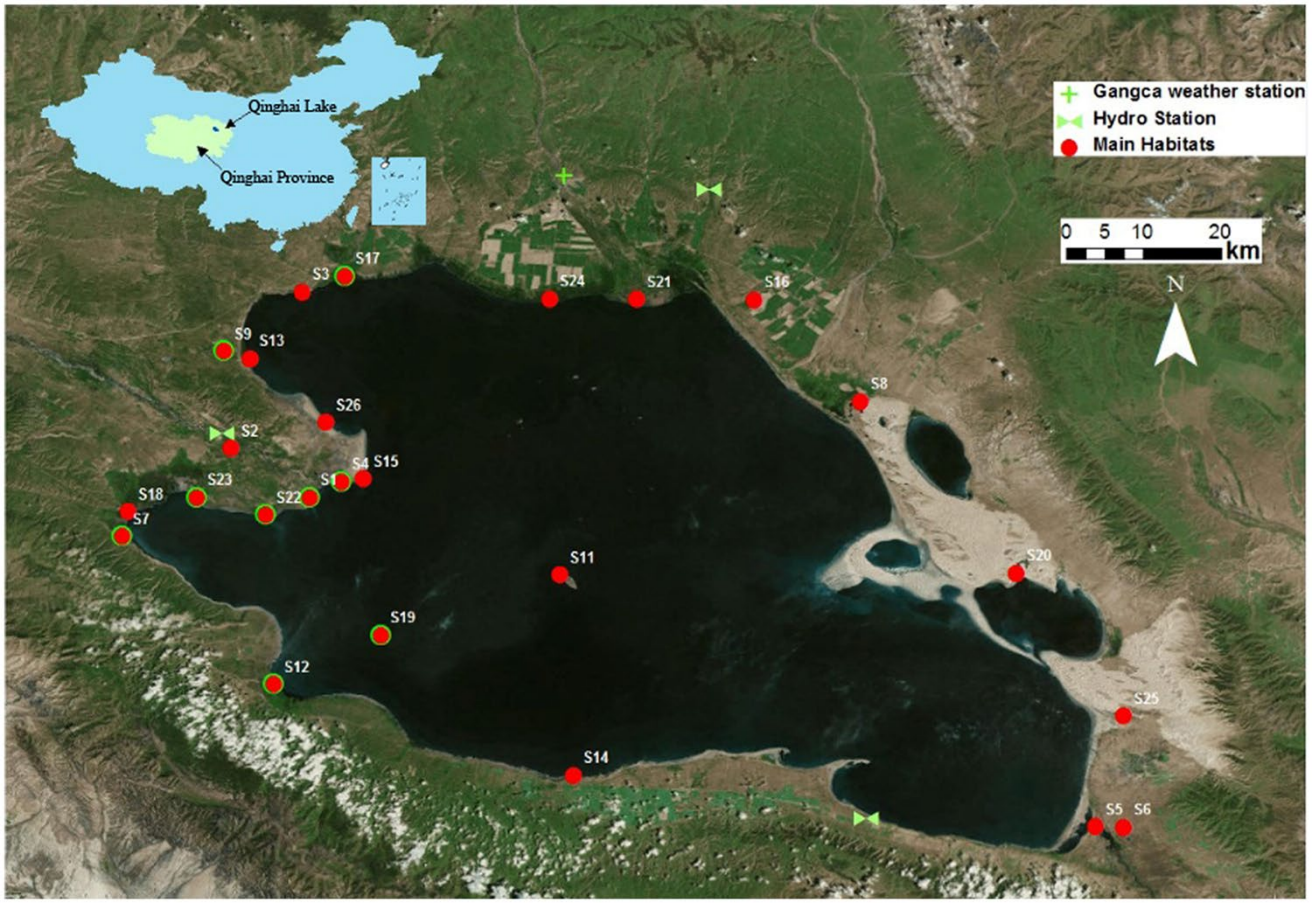
Location of Qinghai Lake and the 24 survey sites. The nine important sites were marked with green rings. Maps were produced using ArcGIS (v 10.2, Esri, Redlands, CA, USA) with World Image (source: Esri, DigitalGlobe, GeoEye, Earthstar Geographics, CNES/Airbus DS, USGS, USDA, AeroGRID, IGN, and the GIS User Community) as background showing the boundary of Qinghai Lake.

Qinghai Lake located at the north-eastern end of the Qinghai-Tibet Plateau (Fig. 6), is the largest extant closed-basin lake in China, with an altitude of 3193 m and an area of c.4300 km^2^. The mean depth of the lake is 21 m (1985) and the maximum depth is 25.5 m^49^. It has a water volume of 7.16 × 10^10^ m^3^ and has a catchment area of about 29 660 km^250^. Qinghai Lake lies in the joint area of the East Asian monsoon, Indian summer monsoon, and Westerly jet stream. The main recharge source direct precipitation and runoff through more than 50 intermittent rivers or brooks^50^. Five permanent rivers (Buha River, Shaliu River, Haergai River, Quanji River, Heima River) provide 80% of total influx^14^. Located in the Central Asian Flyway and East Asian-Australasian Flyway, Qinghai Lake is a vital stopover and corridor for migratory waterfowls. It also provides breeding as well as wintering habitats for hundreds and thousands of birds, with up to 110,000 individuals staging predominantly in its shallower marshes and estuaries. Bar-headed Goose, is one of the flagship species in Qinghai Lake, with a population of above 6000 in summer. Due to its specific ecological character, Qinghai Lake was designated as Ramsar Site in 1992, and it was listed as national nature reserve in 1997.

### Bird Survey

Birds were counted at 24 observation sites (Fig. 6) once each month in April to September from 2007 to 2016. The sites covered five typical habitats: river, estuary, marshland, island and lake. We directly counted all visible Bar-headed Goose using a telescope (Kowa, TSN-820) with a zoom lens of 20–60. We used the peak month (i.e. May) to explore the impacts of environmental factors on Bar-headed Goose population. We chose May based on two considerations. First, the main migration season ends at the end of April (i.e. no more new incomer). Second, the majority of eggs were hatched and chicks have fledged. Therefore, the population is most stable in May.

Of the 24 survey sites, over 85% of the bar-headed geese were observed at the nine areas (marked with a green ring in Fig. 6) located at the western shore of the Lake. Our analysis focused on these nine sites. In addition, the survey data show that two sites (i.e. S04 and S19) have the largest number of birds in March, April and May, and are the main breeding sites in Qinghai Lake area (personnel comm. with staff from the Reserve). We classified the sites as breeding (S04 and S19) and foraging (the others).

### Environmental variables

The long-term climatic data for the period of 1995–2015 was obtained from the nearby Gangca Station (Fig. 6), and total monthly rainfall and mean monthly temperature were calculated for analysis. There are two main tributaries flowing into Qinghai Lake, and the flow rates are gauged (Fig. 6). The sum of the two river flows was analysed as inflow to the Lake. In addition, the daily lake water level is recorded at a hydrological station at Xiashe (Fig. 6).

The satellite-based Normalized Difference Vegetation Index (NDVI) has been the most widely used indicator and proven to be successful to represent vegetation activity among various vegetation indices^51,52^. The time series of NDVI retains information along environmental gradients^53^ as well as the intra- and inter-annual changes in the environment^26,54^. Multi-temporal NDVI has been used to monitor the locations and distributions of land-cover changes^55^; to detect disturbance and quantify patch size^56^; and to predict the waterbird distribution^57^ and abundance^58^. In this study, we created random spatial points within each studied habitat site. The number of points ranged from 12–17 (i.e. covering 75–106.25 ha) depending on the size of the habitat. These points represented about 25% of the foraging and breeding habitats. With the spatial points, we downloaded the 16-day MODIS NDVI products (MOD13Q1) from the U.S. Geological Survey (USGS) Earth Resources Observation and Science Center (EROS, http://LPDAAC.usgs.gov), and calculated the spatial mean monthly NDVI (Feb 2000 to Dec 2016) for each of the nine habitats as an indicator of habitat quality for the herbivore goose.

### Statistical analysis

#### Trends in long-term climate and lake water level

We used the correlated seasonal Mann-Kendall test (CSMK)^28^ to check if there is any significant trend in the time series of climate (rainfall and temperature, 1957–2015), inflow lake water level (1995–2015), and the NDVI (2000–2015). A significant increasing trend in the lake water level might reduce the size of the foraging habitats located the littoral zone of the Lake. The CSMK test is preferred when the seasons are significantly correlated^48^, which is the case for our climatic and hydrological data. We used R package “trend”^59^ for the non-parametric analysis.

#### Modelling the long-term change in spatial mean NDVI

We explored the long-term trend in the monthly NDVI time series, and the impact of environmental factors on its variation with the GAM (Generalized Additive Models) framework^25^. The GAM for time series modelling takes the form:

1 $$g({Y}_{t})={\beta }_{0}+{f}_{seasonal}(month)+{f}_{trend}(time)+f({X}_{t})+{\varepsilon }_{t}$$

where *g* is the link function, *Y*_*t*_ is the response variable (i.e. the spatial mean NDVI in this study); *f*_*seasonal*_(*month*) and *f*_*trend*_(*time*) are smooth functions for the cyclic and trend features we’re interested in; month is the 12 calendar months and time is the number of month since Feb 2000, the start of NDVI time series; *X*_*t*_ are some predictor variables including mean temperature, rainfall and water level in this study; *f*s are the smooth functions that are estimated; and *ε*_*t*_ are independent error terms with a density function described by *N*(*0*, *σ* ^2^).

The general GAM in form (1) assumes that the model errors (residuals) are identically and independently distributed. However, in the case of the time series regression, this assumption is often not fulfilled as the present time series values are often highly correlated with past values for most time series (i.e. autocorrelation; including NDVI time series^57^), therefore, errors of the model will be correlated too. This implies that estimated regression coefficients and residuals of a model are likely to be negatively biased, which also implies that the computed statistical tests or confidence intervals are wrong. To deal with autocorrelation in the NDVI time series, we included an autoregressive model (AR) for errors in our model. Thus, we have the model with the term for errors^59^:

2 $${Y}_{t}=\beta X+{\varepsilon }_{t}\,{\rm{and}}\,{\varepsilon }_{t}=\phi {\varepsilon }_{t-1}+{v}_{t}$$

where equation 2 is a classical AR(1) process, and $\phi$ is an unknown autoregressive coefficient to be estimated.

Model simplification and selection proceeded in an iterative manner, using a multi-model inference approach based on the methods and recommendations of Vatka, *et al*.^40^. First, the full suite of environmental predictors (i.e. temperature, rainfall and water level) and seasonal cycle (month) and trend (time) were included in the model as specified in equations 1 and 2. We then selected which predictors to retain using a more restrictive BIC (Bayesian Information Criterion) with maximum likelihood (ML) parameter estimation. We checked the final model for residual normality and autocorrelation^25^. The GAMs were fit using Gaussian family and an identity link function and residual maximum-likelihood estimator (MGCV version 1.8–7) in R (version 3.10).

#### Modelling Bar-headed Goose population dynamics

To investigate the role of different variables in determining changes in abundance of bar-headed goose, we used general additive mixed models (GAMM) with a negative binomial error structure^60^. GAMM is an extension of GAM by including a random effects term, *Z*_*t*_ *b*, where *Z* is a random effects matrix and *b* is a vector of random effects described by *N*(*0*, *ψ*_*θ*_) with *ψ*_*θ*_ representing a covariance matrix.

3 $$g({Y}_{t})={\beta }_{0}+{f}_{trend}(time)+f({X}_{t})+{Z}_{t}b+{\varepsilon }_{t}$$

As our study focused on May, the peak abundance month, the seasonal cycle in equation 2 was removed. The predictors in equation 3 included mean temperature, rainfall and NDVI in May. Survey site was specified as random effect. In addition, we checked if the bar-head goose population dynamics responded to environmental factors differently at foraging and breeding sites by specifying both a random intercept and random slope term in the GAMM model. Model selection followed the same approaches as the above NDVI time series modelling.

## Electronic supplementary material

Supplementary Figure S1

Supplementary Figure S2

Supplementary Figure S3

Supplementary Table 1

## References

[1] Parmesan C, Yohe G (2003). A globally coherent fingerprint of climate change impacts across natural systems. Nature.

[2] Root TL (2003). Fingerprints of global warming on wild animals and plants. Nature.

[3] Change, I. P. O. C. Climate change 2007: the physical science basis. *Agenda* **6** (2007).

[4] Rinawati F, Stein K, Lindner A (2013). Climate change impacts on biodiversity—the setting of a lingering global crisis. Diversity.

[5] Pachauri, R. K., Allen, M. R. & Meyer, L. Climate Change 2014 Synthesis Report. (The Intergovernmental Panel on Climate Change, Geneva, 2014).

[6] Bellard C, Bertelsmeier C, Leadley P, Thuiller W, Courchamp F (2012). Impacts of climate change on the future of biodiversity. Ecology letters.

[7] Dickey M-H, Gauthier G, Cadieux M-C (2008). Climatic effects on the breeding phenology and reproductive success of an arctic‐nesting goose species. Global Change Biology.

[8] Mclaughlin JF, Hellmann JJ, Boggs CL, Ehrlich PR (2002). Climate change hastens population extinctions. Proceedings of the National Academy of Sciences of the United States of America.

[9] Walther GR (2002). Ecological responses to recent climate change. Nature.

[10] Parmesan, C. Ecological and evolutionary responses to recent climate change. *Annual Review of Ecology*, *Evolution*, *and Systematics*, 637–669 (2006).

[11] Holmes JA, Cook ER, Yang B (2009). Climate change over the past 2000 years in Western China. Quaternary International.

[12] Liu X, Chen B (2000). Climatic warming in the Tibetan Plateau during recent decades. International journal of climatology.

[13] Shi, Y. *Glacial Inventory of China* (*Synthesis volume*) (Science Popularization Press, 2005).

[14] Rhode D (2010). Paleoenvironmental and archaeological investigations at Qinghai Lake, western China: geomorphic and chronometric evidence of lake level history. Quaternary International.

[15] Bishop MA, Yanling S, Zhouma C, Binyuan G (1997). Bar-headed Geese Anser indicus wintering in south-central Tibet. Wildfowl.

[16] Hawkes LA (2011). The trans-Himalayan flights of bar-headed geese (Anser indicus). Proceedings of the National Academy of Sciences.

[17] Johansson US (2007). Build-up of the Himalayan avifauna through immigration: A biogeographical analysis of the Phylloscopus and Seicercus warblers. Evolution.

[18] Fox AD, Madsen J (2017). Threatened species to super-abundance: The unexpected international implications of successful goose conservation. Ambio.

[19] Takekawa J (2009). Geographic variation in Bar-headed Geese Anser indicus: connectivity of wintering areas and breeding grounds across a broad front. Wildfowl.

[20] Chen H, Smith G, Zhang S, Qin K (2005). H5N1 virus outbreak in migratory waterfowl. Nature.

[21] Xu X, Chen H, Levy JK (2008). Spatiotemporal vegetation cover variations in the Qinghai-Tibet Plateau under global climate change. Chinese Science Bulletin.

[22] Wang X (2008). Regional assessment of environmental vulnerability in the Tibetan Plateau: Development and application of a new method. Journal of Arid environments.

[23] Thompson PM, Ollason JC (2001). Lagged effects of ocean climate change on fulmar population dynamics. Nature.

[24] Regehr, E. V., Wilson, R. R., Rode, K. D., Runge, M. C. & Stern, H. L. Harvesting wildlife affected byclimate change: a modelling and management approach for polar bears. J*ournal of Applied Ecology* (2017). 10.1111/1365-2664.12864 PMC5637955 29081540

[25] Wood, S. *Generalized additive models: an introduction with R*. (CRC press, 2006).

[26] Guo W, Ni X, Jing D, Li S (2015). Spatial-temporal patterns of vegetation dynamics and their relationships to climate variations in Qinghai Lake Basin using MODIS time-series data. Journal of Geographical Sciences.

[27] Magnuson JJ (2000). Historical trends in lake and river ice cover in the Northern Hemisphere. Science.

[28] Hirsch RM, Slack JR, Smith RA (1982). Techniques of trend analysis for monthly water quality data. Water resources research.

[29] Zhang G, Xie H, Duan S, Tian M, Yi D (2011). Water level variation of Lake Qinghai from satellite and *in situ* measurements under climate change. Journal of Applied Remote Sensing.

[30] Jiang L, Nielsen K, Andersen OB, Bauer-Gottwein P (2017). Monitoring recent lake level variations on the Tibetan Plateau using CryoSat-2 SARIn mode data. Journal of Hydrology.

[31] Zhang G, Xie H, Kang S, Yi D, Ackley SF (2011). Monitoring lake level changes on the Tibetan Plateau using ICESat altimetry data (2003–2009). Remote Sensing of Environment.

[32] Vázquez, D. P., Gianoli, E., Morris, W. F. & Bozinovic, F. Ecological and evolutionary impacts of changing climatic variability. *Biological Reviews* (2015). 10.1111/brv.12216 26290132

[33] Yao T, Pu J, Lu A, Wang Y, Yu W (2007). Recent glacial retreat and its impact on hydrological processes on the Tibetan Plateau, China, and surrounding regions. Arctic, Antarctic, and Alpine Research.

[34] Piao S, Fang J, He J (2006). Variations in vegetation net primary production in the Qinghai-Xizang Plateau, China, from 1982 to 1999. Climatic Change.

[35] Chen B (2014). The impact of climate change and anthropogenic activities on alpine grassland over the Qinghai-Tibet Plateau. Agricultural and Forest Meteorology.

[36] Coops H, Beklioglu M, Crisman TL (2003). The role of water-level fluctuations in shallow lake ecosystems–workshop conclusions. Hydrobiologia.

[37] Sanz JJ (1996). Effect of food availability on incubation period in the pied flycatcher (Ficedula hypoleuca). The Auk.

[38] Zheng, M. *An introduction to saline Lakes on the Qinghai—Tibet plateau*. Vol. 76 (Springer Science & Business Media, 1997).

[39] Yin F, Deng X, Jin Q, Yuan Y, Zhao C (2014). The impacts of climate change and human activities on grassland productivity in Qinghai Province, China. Frontiers of Earth Science.

[40] Vatka E, Orell M, RytkÖnen S (2011). Warming climate advances breeding and improves synchrony of food demand and food availability in a boreal passerine. Global Change Biology.

[41] Jensen RA (2008). Prediction of the distribution of Arctic‐nesting pink‐footed geese under a warmer climate scenario. Global Change Biology.

[42] Ramanathan V, Feng Y (2009). Air pollution, greenhouse gases and climate change: Global and regional perspectives. Atmospheric Environment.

[43] Martin TE (1987). Food as a limit on breeding birds: a life-history perspective. Annual review of ecology and systematics.

[44] Tremblay I, Thomas DW, Lambrechts MM, Blondel J, Perret P (2003). Variation in Blue Tit breeding performance across gradients in habitat richness. Ecology.

[45] Nilsson J-A (1991). Clutch size determination in the marsh tit (Parus palustris). Ecology.

[46] Martin TE, Li P (1992). Life History Traits of Open‐vs. Cavity‐Nesting Birds. Ecology.

[47] Jones J (2001). Habitat Selection Studies in Avian Ecology: ACritical Review. The auk.

[48] Libiseller C, Grimvall A (2002). Performance of partial Mann–Kendall tests for trend detection in the presence of covariates. Environmetrics.

[49] Li X-Y, Xu H-Y, Sun Y-L, Zhang D-S, Yang Z-P (2007). Lake-level change and water balance analysis at Lake Qinghai, west China during recent decades. Water Resources Management.

[50] Jin Z, You C-F, Wang Y, Shi Y (2010). Hydrological and solute budgets of Lake Qinghai, the largest lake on the Tibetan Plateau. Quaternary International.

[51] Neigh CS, Tucker CJ, Townshend JR (2008). North American vegetation dynamics observed with multi-resolution satellite data. Remote Sensing of Environment.

[52] Piao, S. *et al*. Evidence for a weakening relationship between interannual temperature variability and northern vegetation activity. *Nature communications* **5** (2014). 10.1038/ncomms6018 25318638

[53] Pettorelli N (2011). The Normalized Difference Vegetation Index (NDVI): unforeseen successes in animal ecology. Climate Research.

[54] Davis SE, Nager RG, Furness RW (2005). Food availability affects adult survival as well as breeding success of parasitic jaegers. Ecology.

[55] Lunetta RS, Knight JF, Ediriwickrema J, Lyon JG, Worthy LD (2006). Land-cover change detection using multi-temporal MODIS NDVI data. Remote sensing of environment.

[56] Jin S, Sader SA (2005). MODIS time-series imagery for forest disturbance detection and quantification of patch size effects. Remote Sensing of Environment.

[57] Wen L, Saintilan N, Reid JR, Colloff MJ (2016). Changes in distribution of waterbirds following prolonged drought reflect habitat availability in coastal and inland regions. Ecology and evolution.

[58] Guan L (2016). Optimizing the timing of water level recession for conservation of wintering geese in Dongting Lake, China. Ecological Engineering.

[59] Pohlert, T. Trend: Non-ParametricTrend Tests and Change-Point Detection. *R package version 0*.*0*, **1** (2015).

[60] Wen L, Rogers K, Saintilan N, Ling J (2011). The influences of climate and hydrology on population dynamics of waterbirds in the lower Murrumbidgee River floodplains in Southeast Australia: implications for environmental water management. Ecological Modelling.

