# Supplementary material for: Productivity benefits of warming at regional scale could be offset by detrimental impacts on site level hydrology
Source: Sci Rep. 2017 Nov 9;7:15144. doi: 10.1038/s41598-017-15136-8 (PMC5680237; doi:10.1038/s41598-017-15136-8)
Supplement: Supplementary file 3 — Supplementary Figure S3 [file 41598_2017_15136_MOESM3_ESM.doc]

**Productivity benefits of warming at regional scale could be offset by detrimental impacts on site level hydrology**

Qing Zeng ^1*^, Yamian Zhang [[1]](#footnote-2), Li Wen ^1,2^, Zhaxijie Li ^1,3^, Hairui Duo ^1^, and Guangchun Lei ^1^


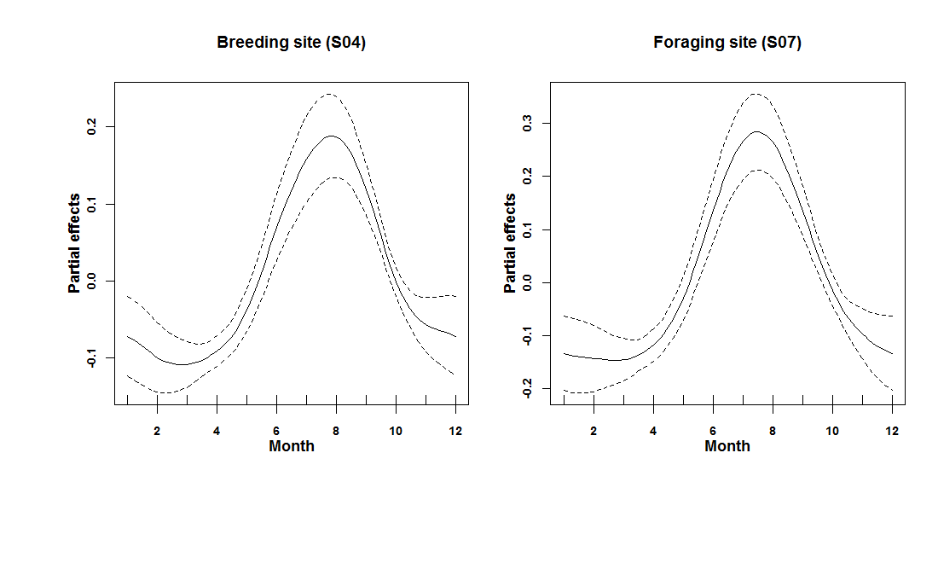


Supplementary Figure S3 The seasonal cycles of NDVI (mean and ±SE) in breeding (S04) and foraging (S07) sites in Qinghai Lake modelled using GAMM.

1. 1 School of Nature Conservation, Beijing Forestry University, Beijing, China. 2 Science Division, Office of Environment and Heritage, Sydney, New South Wales, Australia. 3 Tibet Museum of Natural Science. ∗These authors contributed equally to this work. Correspondence and requests for materials should be addressed to G.L. (email: guangchun8099@gmail.com) or L.W. (email: li.wen@environment.nsw.gov.au) [↑](#footnote-ref-2)
