# Supplementary material for: Productivity benefits of warming at regional scale could be offset by detrimental impacts on site level hydrology
Source: Sci Rep. 2017 Nov 9;7:15144. doi: 10.1038/s41598-017-15136-8 (PMC5680237; doi:10.1038/s41598-017-15136-8)
Supplement: Supplementary file 4 — Supplementary Table 1 [file 41598_2017_15136_MOESM4_ESM.doc]

**Productivity benefits of warming at regional scale could be offset by detrimental impacts on site level hydrology**

Qing Zeng ^1*^, Yamian Zhang [[1]](#footnote-2), Li Wen ^1,2^, Zhaxijie Li ^1,3^, Hairui Duo ^1^, and Guangchun Lei ^1^

Supplementary Table 1 Summary of the final GAMM for bar-headed goose population in the main habitats of Qinghai Lake

|  | EFD | λ^2^ | p |
| --- | --- | --- | --- |
| s(Site) | 5.270 | 24.46 | 0.000 |
| s(Type, Time) | 4.662 | 636.59 | 0.001 |
| s(Type, NDVI) | 0.867 | 13.81 | 0.048 |

1. EFD = estimated degree of freedom, s = smooth function.
2. The final model has an adjusted R^2^ 0.898, and explained 67.4% in abundance deviance.

1. 1 School of Nature Conservation, Beijing Forestry University, Beijing, China. 2 Science Division, Office of Environment and Heritage, Sydney, New South Wales, Australia. 3 Tibet Museum of Natural Science. ∗These authors contributed equally to this work. Correspondence and requests for materials should be addressed to G.L. (email: guangchun8099@gmail.com) or L.W. (email: li.wen@environment.nsw.gov.au) [↑](#footnote-ref-2)
